# Transculturality: The New Frontier of Care Relationships

**Published:** 2012-10-11

**Authors:** Roberta T. Di Rosa

**Affiliations:** Università di Palermo

**Keywords:** Transculturality, aid relationship, migrants

## Abstract

Taking care of migrants constitutes a new challenge for the actual operative structures of the services in general and for the sanitary service in particular, requiring a global and permanent rethinking with regards to both the offer and the procedures for decoding the requests. What determines the complexity of offering care while respecting differences is the fact that it can not be done without the professionals individually deconstructing racism and maturing an anti-racist awareness. However, attention to this question is neither widespread nor shared during the training of doctors and of health service workers in general. It is necessary, therefore, to broaden the traditional staff-client relationship (usually articulated in the dyad staff-subject/client-object) until it is recognized that both parts have a double role, both as a subject and as an object, within the aid process. The transcultural model is based on the concept of reciprocity. What the transcultural relationship involves is a parallel process of a redefinition of identity, both of the doctor or health service worker and of the client: it is necessary for both to question parameters that they considered certain, overcoming their inevitable resistance in the process. It appears necessary to explore within the training programs the strategies that people, in this specific case the professionals whose work regards health, adopt to avoid challenging racism and the implications that these can have in their daily duties.

## I. INTRODUCTION

Taking care of migrants constitutes a new challenge for the actual operative structures of the services in general and for the sanitary service in particular, requiring a global and permanent rethinking with regards to both the offer and the procedures for decoding the requests.

What determines the complexity of offering care while respecting differences is the fact that it can not be done before the professionals individually deconstructing racism and maturing an anti-racist awareness. However, attention to this question is neither widespread nor shared during the training of doctors and of health service workers in general. Nor do ordinary training courses exist that deal with the recognition and management (or prevention) of discrimination and/or racism in its various forms.

In practice it can be seen how in everyday contact with Others the habitual conventions of respect and equality take on varying shades until they manage to constitute deep and serious exceptions between one individual and another, both in the access to services and in their application, depending on nationality or the colour of one’s skin. In the services, the staff who are continually exposed to contact with such a variegated clientele, one which is difficult both to understand and to deal with, run an increasingly higher risk of unconsciously developing attitudes that are improper from an ethical and professional point of view, such as xenophobia, uncritical reception or charitable paternalism.

The distance, which is a consequence of professional difficulty, increases exponentially where personal dynamics intervene: the collective imagination comes into play, altering even the perception of one’s own cultural identity and that of others, creating disorientation, distrust, at times fear: “When we are faced with a foreigner perhaps there is the proof that he possesses an attribute that makes him different from us, from the members of the category to which he presumably should be a part (….) in this way, in our mind he becomes declassed from being a complete person to whom we are commonly accustomed to, to being a marked or disreputable person. This attribute is a stigma, especially when it has the ability to effect a deep sense of disrepute.”^1^

Among doctors, nurses or whoever finds himself having to decode information which is communicated in a defective way from a linguistic point of view and which in any case is difficult to understand because it makes reference to symbolic systems that are distant and often unknown, there is a resistance to “listening” to information that makes one question one’s own values, incredulity when faced with unknown facts, the struggle that has to be faced with one’s own prejudices, having to accept the competence of others who have different knowledge; all this can interfere with listening, with understanding the discomfort, the perception of the level of suffering of the immigrant and therefore with the possibility of a correct interpretation and a relationship with the patient that guarantees his compliance with the indications received.

Equally at risk, although in an opposite sense, is an unbalanced approach to cultural difference that, as Mazzetti underlines: “exaggerated in our perception, it often leads us to forget that we have before us a person and not a culture, and, as a result, to interpret the reactions of this person as a cultural trait, as if every individual were an archetype of the world from which he comes from.” i

It is not infrequent in our society to believe that racism is a social problem that requires government intervention, rather than one of the principal preoccupations in the behaviour of individuals both as people and as professionals.

In the same way, it is not infrequent when dealing with foreign patients for there to be an emphasis on being “culturally attentive”, which more often than not fails: being considered “cultural expressions” mostly provokes in the clients the perception of a lack of recognition of the singularity of their story, that their personal subjectivity as recounted to the service is not understood.

Both the operator and the consumer sense the difficulties and the risks inherent in a relationship involving intercultural aid – effectively examined by Mazzettiii – which consist both in overestimating and in underestimating the cultural difference. In the first case, classifying everything that is not understood or shared as “cultural” often leads to a depersonalization of the interlocutor, who is no longer received as a unique individual but as a stereotypical representative of a cultural group; in the second case, it is preferable to ignore or to deny the cultural specificities of the environment in which a person has structured his identity, in the name of a universalism that often borders on ethnocentrism, considering valid and applicable to everyone the values, the customs and the priorities of one’s own cultural system. If one keeps a balanced approach as much as possible, one feels the need to continuously redefine one’s professional identity - or, according to Laiiii, one’s disidentity - experimenting relational styles that are different from the usual, trying to acquire new resources and competences.

It is not simple for anyone to admit that everyone harbours prejudices and stereotypes in their minds and that nobody is immune to these. To realise this it is often necessary to come up against it through one’s professional work, or in one’s personal life, with a case that involves key elements such as religion, politics, culture, gender, relationships, to realize how difficult it is to practice one’s professional role with the necessary detachment and openness.

Recognizing the differences and identifying the similarities, bearing in mind the danger that the differences become generalized as stereotypes, is only the first step. As Clara Gallini says, it is necessary not to deny one’s specificity, but to live it in a critical way: “paraphrasing the image of the “burden of the white man” we could say that within this burden we carry a cultural inheritance that – for better or for worse - can not be realistically rejected en masse, also because with it we would lose, together with our memories, also the tools of remembrance and communication. In this sense De Martino also proposed the perspective not of radical abandonment, but of exercise of what he called “critical ethnocentrism”, which is also a pedagogy of ourselves”iv. This would allow, as Colasanti and Geraciv mantain, to know how to complete the passage from the observation/exploration of the other – which is always somewhat ethnocentric, typical of western culture - to the analysis of the relationship between staff worker/user, in which both are object of an investigation of their interaction and at the same time the principal subject responsible for the interaction and for its results.

It is necessary, therefore, to broaden the traditional staff-client relationship (usually articulated in the dyad staff-subject/client-object) until it is recognized that both parts have a double role, both as a subject and as an object, within the aid process. An effective aid relationship, constituted by the intersection of these two paths, will know how to hold in consideration both the cultural background of the parts involved and their specific individuality.

## II. THE TRANSCULTURAL AID RELATIONSHIP

A valid alternative is “transcultural dialogue”, a work methodology that is able to provide adequate responses to the needs placed by modern society and thus by the meeting of cultures. The definition that better describes an aid relationship between individuals belonging to different cultures is of it being both an intercultural relationship (a term which is however misused in the offer of reception services) and a transcultural one. “The first means having different traditions that look at each other, listen to each other, exchange something, starting from the mutual differences, therefore beginning with distances of a religious, spiritual, philosophical, or artistic nature. Transculturality looks for what can make people closer, notwithstanding these differences [...] Thus not only the differences, not the distances, because if we emphasize an attention for the differences, we run the risk of confirming the mutual distances. I believe instead that it is important to individualize the mutual contiguities or the communities, to give a contribution to the demolition of the prejudices that are always present within us and which inhabit our every reflection.” vi

The transcultural model is based on the concept of reciprocity, which substantiates itself in the recognition and valorisation of human capital, the consideration of the patient as a “subject” of the relationship who is able to produce change, the conception of differences as a resource and not as an obstacle. What the transcultural relationship involves is a parallel process of a redefinition of identity, both of the doctor or health service worker and of the client: it is necessary for both to question parameters that they considered certain, overcoming their inevitable resistance in the process.

It is clear, but not guaranteed, that this also requires that the patient is disposed and able to interact in an open and accessible way in order to explore the common ground that is found at an intermediate point of the path with respect to one’s starting point. Recognizing a dynamic of mutual discovery and parallel change means to give attention to what is happening in the here and now of the dialogue, beyond the cultural differences, re-establishing a position of equal dignity between the interlocutors, who collaborate to find the most effective relationship to attain success in the aid process.

The change that aid professionals requires is on two levels, as Bennetvii describes: at the mindset level, or, rather, as a set of attitudes and vision of the world, a condition that has at its base the acceptance of the recognition of differences and the keeping of a positive attitude toward these; and at a skill level, that is, as a set of competences and practical knowledge which are necessary to deal with any intercultural meeting, with the ability to use some general theoretical-practical frameworks to learn how to learn in intercultural situations.

For there to be effective interaction in diversity it is essential to accept the differences that characterize the subjects, bring the initial cognitive hypotheses to awareness, know the cultural frameworks of reference, observe, listen and know how to ask questions that are useful for understanding the other’s real perspective.

An effective diagnostic and care process is one in which a path leading to cultural decentralization is also established, that is to say, one uses the capacity not to judge the cultural elements that emerge, to acknowledge which are our references and not prefer them to the knowledge and understanding of the other, to open a space for the narration and the expression of the cultural references, to perceive which are the cultural counter-attitudes that create barriers between the interlocutors.

## III. PREPARING THE CHANGE: CONTINUOUS TRAINING

Being constantly balanced between differentialism and universalism requires therefore that the sanitary services (and not just them) not only equip themselves with cultural mediators who can provide linguistic and cultural competence, but also, and above all, to start refresher and training courses on the “relational competences” of the staff that contain paths to personal awareness on one’s own prejudices, on their expectations towards the patients (at times marked by a certain exoticism), on what ground can be realistically shared. In this “meeting between people”, they encounter reciprocal difficulties deriving from the fact that, both for the patients and for the staff, culture determines what they perceive as a problem, how they express it, who can intervene to help, what types of solutions can be taken into consideration.

What should be underlined is the importance of acquiring ever greater means for understanding and interpreting the ethno-cultural identity (one’s own as well as that of others) and how they influence the behaviour of individuals, to avoid being influenced by stereotypes. As we know, generalizations have an important function in allowing us to take action and also to explain the unknown to us. If they remain the same, however, they replace accurate descriptions of new situations, observations and analysis that add knowledge. It is also common knowledge that they have an important role in reinforcing racism. Even if the concept of race has been contested and generally overcome by a good part of the scientific worldviii, this has not been enough to make us immune from developing prejudices, both at a personal level, and, even worse, at a community one.

Self-conscience (intended as a process that individuals use for making connections between the social relationships that they themselves perpetuate through their attitudes, values and behaviours and the social position that they have) is an essential characteristic of a society that wants to oppose racism. It appears necessary to explore within the training programs the strategies that people, in this specific case the professionals whose work regards health, adopt to avoid challenging racism and the implications that these can have in their daily duties.

To improve a competence suitable at both levels requires both a requalification of university education and a greater care in refresher courses and in continuous training. The inclusion of courses that construct competences that allow us to discover and reveal prejudices within us which affect our actions and our choices can be a form of protection, balance and distance from our presumed certainties.
